# Supplementary material for: Identification and functional analysis of non-coding regulatory small RNA FenSr3 in Bacillus amyloliquefaciens LPB-18
Source: PeerJ. 2023 May 15;11:e15236. doi: 10.7717/peerj.15236 (PMC10194069; doi:10.7717/peerj.15236)
Supplement: Supplemental Information 4 [file peerj-11-15236-s004.zip › KO/CK-vs-T1_map/map05418.html]

KEGG PATHWAY: Fluid shear stress and atherosclerosis - Reference pathway


|  |  |
| --- | --- |
| **Fluid shear stress and atherosclerosis - Reference pathway** |  |

[
Pathway menu
| Organism menu
| Pathway entry
| Show description
| User data mapping
]

Shear stress represents the frictional force that the flow of blood exerts at the endothelial surface of the vessel wall and plays a central role in vascular biology and contributes to the progress of atherosclerosis. Sustained laminar flow with high shear stress upregulates expressions of endothelial cell (EC) genes and proteins that are protective against atherosclerosis. The key shear stress-induced transcription factors that govern the expression of these genes are Kruppel-like factor 2 (KLF2) and nuclear factor erythroid 2-like 2 (Nrf2). On the other hand, disturbed flow with associated reciprocating, low shear stress generally upregulates the EC genes and proteins that promote oxidative and inflammatory states in the artery wall, resulting in atherogenesis. Important transcriptional events that reflect this condition of ECs in disturbed flow include the activation of activator protein 1 (AP-1) and nuclear factor kappaB (NF-kappaB).

|  |  |  |
| --- | --- | --- |
| Reference pathway | 184% 150% 122% 100% 82% 67% 55% | 图片下载 |
